# About the Toxicity of Muscular “Autolysats” in Cases of Shock

**Published:** 1919-01

**Authors:** 


***About the Toxicity of Muscular "Autolysats" in Cases of Shock.*** By MM. PIERRE DELBET and KARAJONOPOULOS. *Bulletin de l'Académie de Médecine de Paris*, July 2, 1918.

Some experiments were undertaken by the authors in order to determine the "role" of intoxication in traumatic shock. They consider this as very important, the toxicity of bruised tissues being now a well known fact.

Without the interference of any bacilli the bruised tissues acquire spontaneously a high toxic power, and this may develop in the organism precisely the characteristic features of shock.

Tissues of dead animals were put aside immediately after death had occured, finely lacerated, and kept in normal saline solution at 37° C. After some time the whole was filtrated through a thin gauze, and the fluid part, containing the "autolysats", injected into the peritoneum of an animal of the same variety.

Tests were positive with guinea-pigs and rats, negative with dogs, according to the impossibility of obtaining aseptic "autolyse".

The "autolysats" obtained from tissues of grey rats proved very toxic especially if the animals had been fed with much meat.

A few seconds after the infection, the animal would collapse into coma, the rate of breath increasing to 140 a minute; then it would become quite unconscious; gradually the rate of breath would fall to 40 and even 30 a minute, and the animal would soon die.

Among 22 rats injected, 2 healed and 20 died, after intervals of from 5 minutes to 20 hours. Intoxication of the nervous system seems responsible for cases of sudden death.

The reaction to the toxic power of these autogenous toxins is essentially different according to each animal.

A certain dose of toxin seems ess dangerous if previously diluted.

The authors consider that the symptomatic treatment should be associated with a pathogenic treatment in cases of shock; the toxic focus should be removed as soon as possible, either by amputation, if the limb seems helplessly ruined, or by thorough resection of all bruised tissues. This should be done while the patient is still suffering from all shock symptoms.

